# Supplementary material for: Content-rich biological network constructed by mining PubMed abstracts
Source: BMC Bioinformatics. 2004 Oct 8;5:147. doi: 10.1186/1471-2105-5-147 (PMC528731; doi:10.1186/1471-2105-5-147)
Supplement: Additional File 2 — The original results of the above study (non-essential files are deleted to keep the file size under the limit set by BMC bioinformatics). [file 1471-2105-5-147-S2.bz2 › chilibotAdditionalFile2/dip05/407ID11468284E10068/html/CSF2_CSF3R.html]

 


 **CSF2** and **CSF3R** 
  
Found 185 abstracts in PubMed, retrieved 05.  
 

 What does Google say? 
 PDF only 
| .edu only 

---

- Rev Physiol Biochem Pharmacol< MedlineTA>Rev Physiol Biochem Pharmacol,   **Granulocyte colony stimulating factor **[CSF2]** and its receptor in normal hematopoietic cell development and myeloid disease.** .
  Hematopoiesis, the process of blood cell formation, is orchestrated by cytokines and growth factors that stimulate the expansion of different progenitor cell subsets and regulate their survival and differentiation into mature blood cells.
  Granulocyte colony stimulating factor **[CSF2]** G CSF is the major hematopoietic growth factor involved in the control of neutrophil development.
  G CSF is now applied on a routine basis in the clinic for treatment of congenital and acquired neutropenias.
  G CSF activates a receptor of the hematopoietin receptor superfamily, the G CSF receptor G CSF R **[CSF3R]** , which subsequently triggers multiple signaling mechanisms.
  Here we review how these mechanisms contribute to the specific responses of hematopoietic cells to G CSF and how perturbations in the function of the G CSF R **[CSF3R]** are implicated in various types of myeloid disease.
